# Prosthetic Rehabilitation of a Post-mucormycosis Maxillectomy Defect Using a Maxillary Hollow Obturator and Complete Denture: A Case Report

**DOI:** 10.7759/cureus.83881

**Published:** 2025-05-11

**Authors:** Shubham K Srivastava, Shitij Srivastava, Abhinav Shekhar, Debajyoti Sarkar, Abhishek Singh

**Affiliations:** 1 Department of Prosthodontics, Sardar Patel Post Graduate Institute of Dental and Medical Sciences, Lucknow, IND

**Keywords:** complete denture, maxillofacial prosthesis, mucormycosis, palatal obturator, quality of life

## Abstract

Mucormycosis is an opportunistic fungal infection that severely impacts immunocompromised patients. Invasive mucormycosis tends to require surgical procedures like maxillectomy, leading to a complex oro-nasal communication and a severe compromise of oral function, speech, and esthetics. The following case report describes the prosthetic rehabilitation of a patient after maxillectomy post-mucormycosis. A maxillary complete denture with a closed hollow bulb obturator was constructed to restore oral function, facilitate speech, and improve facial esthetics. The impression-making technique was meticulously altered to obtain a precise recording of the defect, and a new flasking technique was used during processing to create a lightweight yet retentive prosthesis. Thermocol was strategically utilized to hollow the obturator and alveolar ridge segments, thereby reducing the weight while preserving structural support. Soft relining with Molloplast B (Detax GmbH & Co. KG, Ettlingen, Germany) was performed to enhance mucosal adaptation as well as patient comfort. After-insertion assessment indicated optimal fit, retention, and patient satisfaction, with marked improvement in speech and mastication. Follow-up at regular intervals showed maintained function and comfort, underlining the critical role of prosthodontic rehabilitation in the interdisciplinary management of post-mucormycosis maxillary defects.

## Introduction

Mucormycosis is a rapidly progressing, potentially life-threatening opportunistic fungal infection caused by fungi from the order of Mucorales, most commonly of the Rhizopus species. It shows a predominance among immunocompromised individuals, that is, uncontrolled diabetic patients with hematologic malignancies and those treated with corticosteroids. Well-documented risk factors include uncontrolled diabetes mellitus, particularly in the presence of diabetic ketoacidosis; hematologic and solid organ malignancies, prolonged corticosteroid use, neutropenia, immunosuppression following hematopoietic stem cell or solid organ transplantation, and, more recently, coronavirus disease 2019 (COVID-19), which has been increasingly associated with secondary invasive fungal infections [1]. A multicenter retrospective study conducted across India reported a significant rise in mucormycosis cases during the COVID-19 pandemic. Specifically, there was a 2.1-fold increase in cases during September-December 2020 compared to the same period in 2019. This surge is attributed to factors such as uncontrolled diabetes mellitus, widespread corticosteroid use, and COVID-19-induced immunosuppression [2].

Rhino-orbito-cerebral mucormycosis is the most frequent type, and it usually warrants drastic surgical measures, such as partial and total maxillectomy, to contain the infection. These surgical defects cause drastic functional and psychological impairments due to oro-nasal communication affecting speech, mastication, and swallowing, and facial aesthetics [3].

Prosthetic rehabilitation plays a crucial role in restoring oral functions and quality of life in affected persons. Fitting a lightweight, retentive, and functional hollow bulb obturator for maxillary defects in conjunction with a complete denture will improve the quality of life for the patient. Impression and flasking techniques would also be of great importance in accurately capturing the defect morphology and reducing the weight of the prosthesis [4-5].

This case report demonstrates the prosthetic management of a post-mucormycosis maxillectomy defect. The report would authenticate the fabrication of a maxillary closed hollow bulb obturator with a complete denture.

## Case presentation

A 55-year-old male was referred to the Department of Prosthodontics for prosthetic rehabilitation post-maxillectomy due to mucormycosis. The patient had a history of significant underlying comorbidities, including poorly controlled diabetes mellitus, and was subsequently diagnosed with chronic invasive fungal rhinosinusitis, which progressed to mucormycosis.

**Figure 1:**
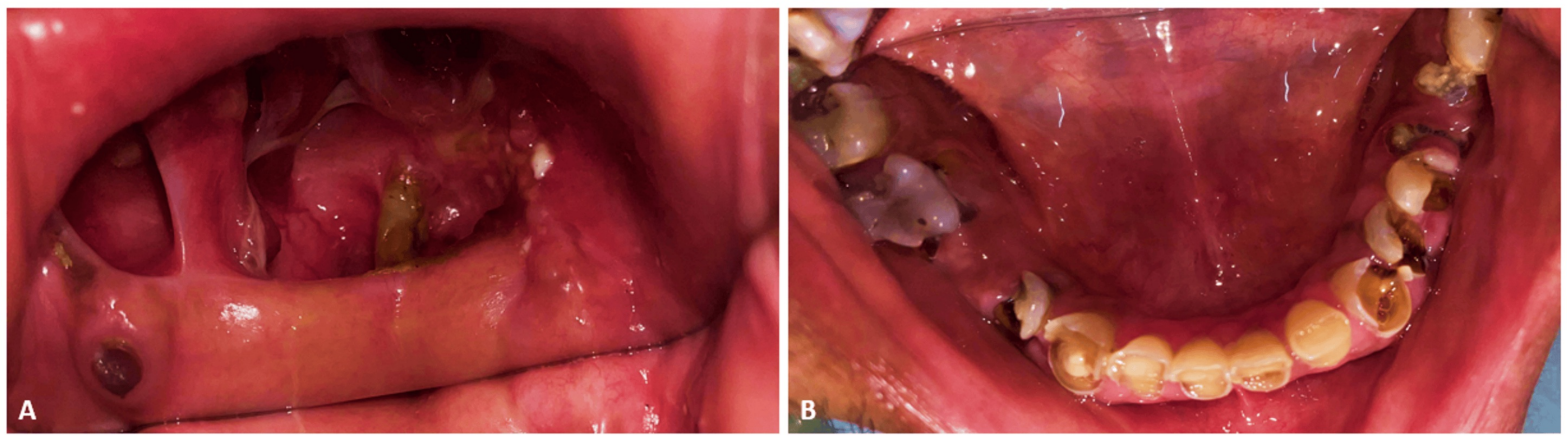
Intraoral photographs A: Maxillary arch demonstrating postoperative healing following maxillectomy; B: Mandibular arch showing multiple grossly decayed teeth with extensive carious destruction

After confirming the diagnosis of mucormycosis by diagnostic nasal endoscopy (DNE) and radiographic imaging, the patient underwent subtotal maxillectomy under general anesthesia to remove necrosed maxillary bone along with infected tissue. The defect was classified as Brown's Class IIb maxillary, involving an anterior cross-section of the hard palate without orbital involvement [6]. Aramany was not appropriate since the maxilla was left nearly edentulous postoperatively, and only a root stump of the maxillary third molar was found remaining in the first quadrant. Examination of the mandibular arch shows severely attrited and periodontally compromised teeth; however, the patient declined any treatment for the lower arch (Figure 1).

**Figure 2:**
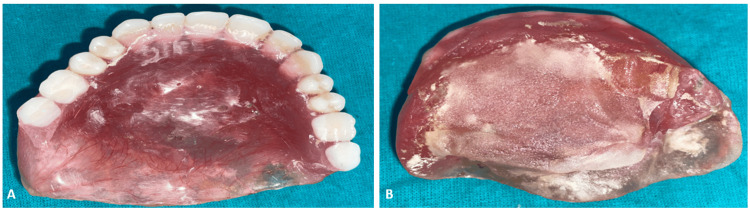
Ill-fitting complete denture A: Cameo-surface; B: Intaglio-surface

His previous denture had been worsening his problems with speech, deglutition, and mastication owing to the large oro-nasal communication. After extensive evaluation and detailed discussion with the patient, a lightweight, hollow closed-bulb maxillary obturator was proposed as a treatment plan to restore these vital oral functions along with improvement in esthetics and comfort. It was designed to achieve maximum retention and stability, leading to overall improved quality of life for the patient after surgery (Figure 2).

**Figure 3:**
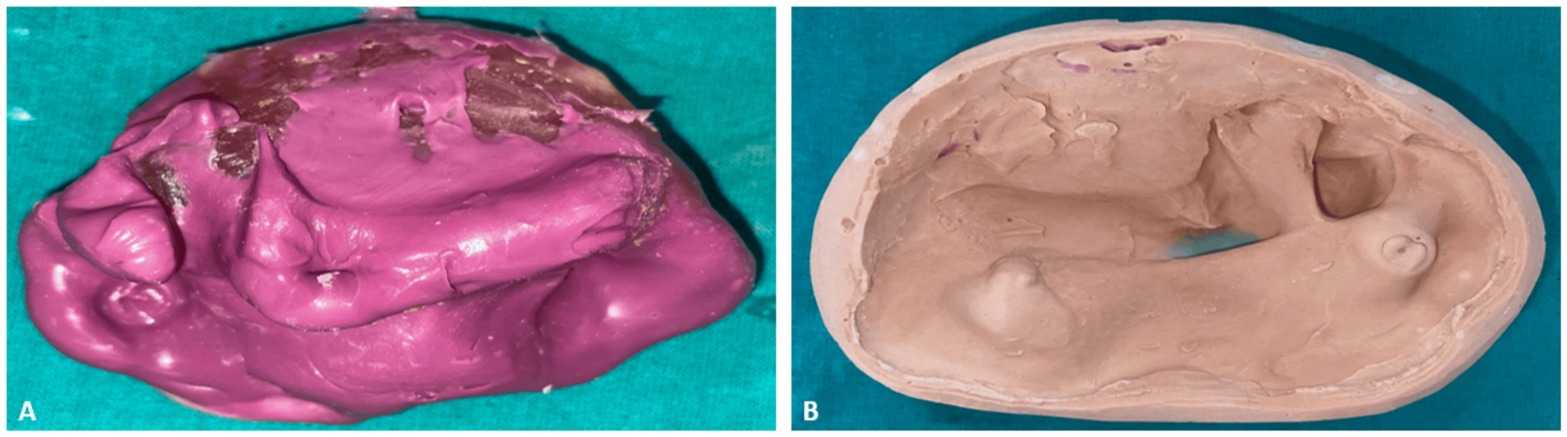
Primary impression and cast A: Primary impression done using monophase impression material; B: The primary cast was obtained using die stone (type IV), and the base was prepared using dental stone (type III)

The preliminary impression was made using a hydrophilic, addition-cured silicone polyvinyl siloxane, Dentsply Aquasil Ultra Impression Material (Dentsply Sirona, York, PA, USA), on the existing complete denture. The primary cast was then obtained by pouring the impression with Type IV dental stone (Elite Rock, Zhermack SpA, Badia Polesine, Italy), while a base was prepared using Type III dental stone (Kalstone Green, Kalabhai Karson Pvt. Ltd., Mumbai, India) to provide structural support for the cast during jaw relation recording and further laboratory procedures (Figure 3).

**Figure 4:**
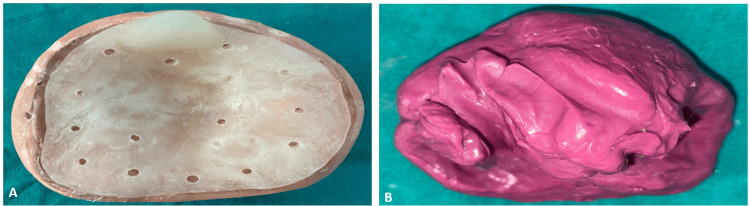
Custom tray and final impression A: Fabrication of a custom tray with multiple holes; B: Final impression using monophase impression material

The custom tray was then made on the primary cast with a self-cure clear acrylic resin (Self-Cure Denture Base Acrylic Material, Pyrax Polymers, India). To improve the mechanical retention of the impression material and provide stability during the impression process, multiple holes were provided in the tray. The final impression was made with a hydrophilic, addition-cured silicone polyvinyl siloxane, Dentsply Aquasil Ultra Impression Material (Dentsply Sirona, York, PA, USA), known for capturing the fine details, including very minute characteristics of the defect, thus ensuring more accuracy for further prosthetic design and fabrication stages (Figure 4).

**Figure 5:**
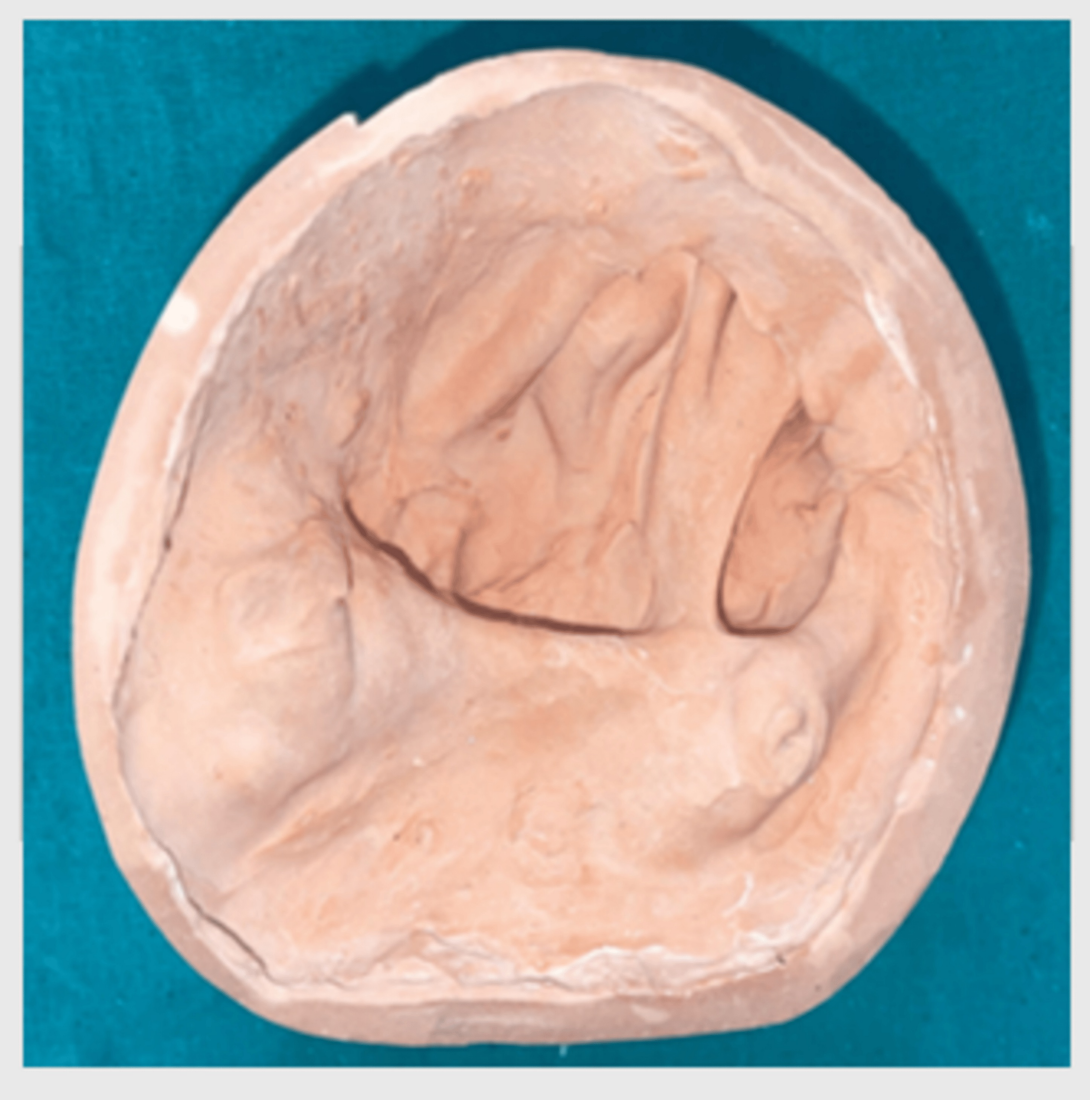
Master cast

The finished impression was poured with Type IV dental stone (Elite Rock) to make the master cast, and the cast created through this method is fundamental for the fabrication of a definitive prosthesis to ensure the fit and aesthetics of the final restoration are accurate (Figure 5).

**Figure 6:**
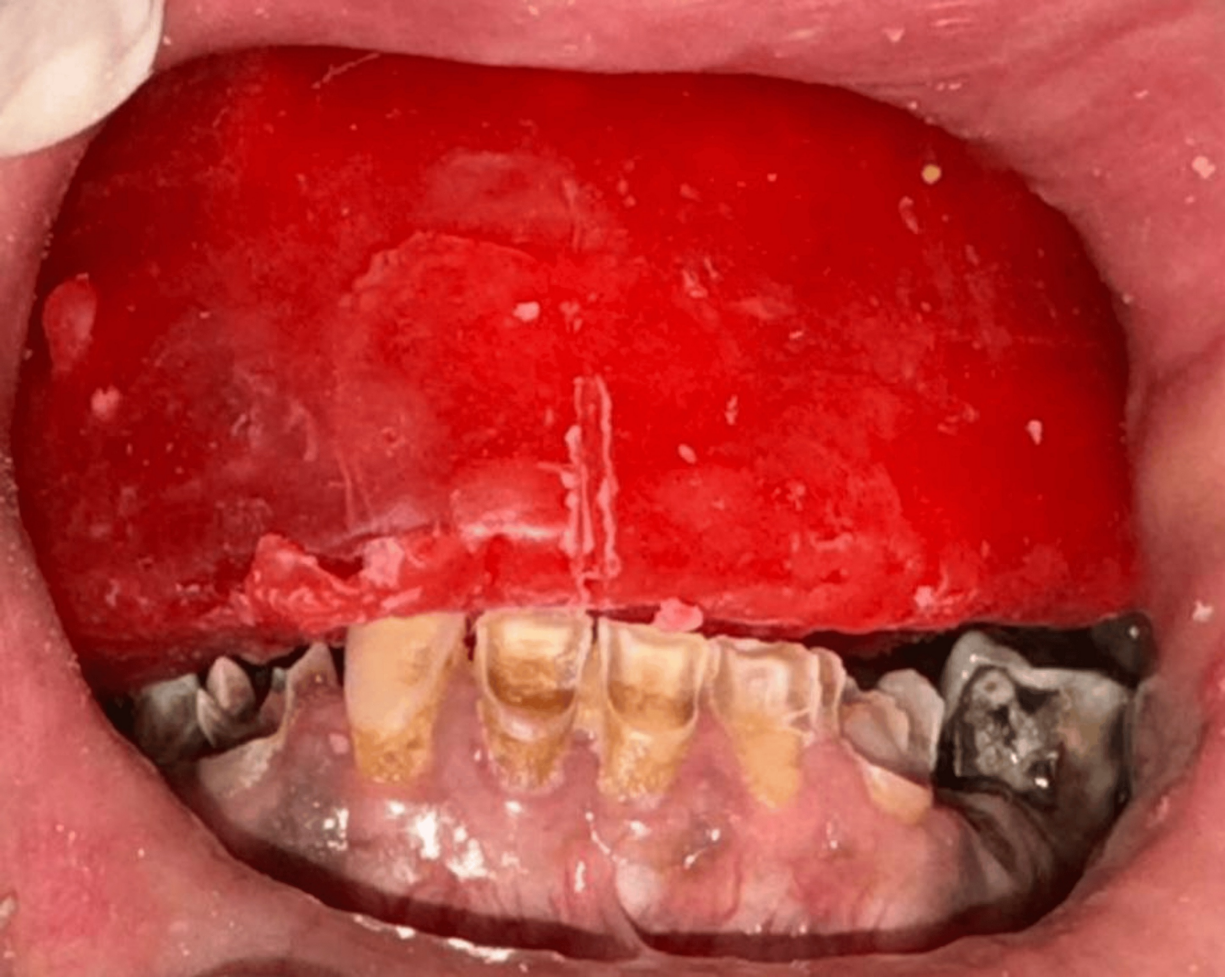
Jaw relation registration Vertical dimension and centric relation recorded by using occlusal rims.

Any undercuts on the master cast were carefully blocked out using Geo Block-out Wax (Renfert, Hilzingen, Germany), and the denture base was then fabricated using self-cure acrylic resin. The occlusal rim was fabricated using Dental Modelling Wax (Pyrax Polymers), providing a stable structure for occlusal registration and subsequent adjustment of vertical dimension and centric relation (Figure 6).

The case was then mounted on a semi-adjustable articulator after jaw relation registration to simulate, as closely as possible, the functional occlusion of the patient. This particular step makes it easier to adjust the maxilla-mandibular relation to simulate patient-actual jaw movements, followed by the careful arrangement of the teeth on the denture base as per the planned optimal occlusion.

**Figure 7:**
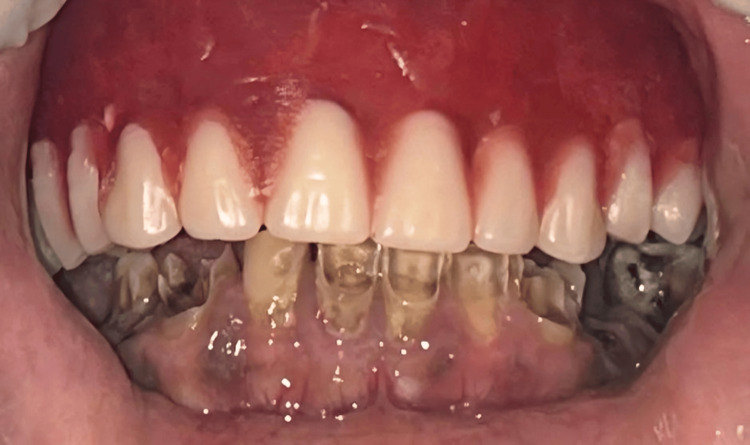
Try-in stage

It was, as per patient preference, the lower arch that was not planned for rehabilitation at this stage. Though this was done, certain considerations were made in maintaining the occlusal plane during teeth arrangement to enable plausible later rehabilitation of the lower arch. The mandibular worn-out teeth were prepared and adjusted to facilitate an accurate and functional occlusion. This was done to optimize the occlusal relationship and ensure proper alignment with the maxillary prosthesis. Therefore, vertical dimension and occlusal relationship were shaped to enable simple integration of the lower arch prosthesis once the patient decides to rehabilitate it sometime in the future (Figure 7).

After completion of the try-in stage, the final processing of the maxillary closed hollow bulb obturator with complete denture was done through acrylization. 

In the first flasking, the setup, including the teeth arrangement, was put into a flask and filled with dental stone for molding. After setting the mold, dewaxing was done to burn away the wax, hence leaving it only for acrylic resin to penetrate the mold. Heat-cured acrylic was packed carefully into the empty mold to cover the base and the teeth arrangement entirely, without trapping air bubbles or voids.

To make it a hollow denture, the obturator was hollowed out during the packing stage. Since the defect was very large and to make the prosthesis lightweight, thermocol was placed between the acrylic at the defect site and the alveolar ridge part.

Resin packed into the flask was now put in a curing unit and subjected to heat and pressure for polymerization of the acrylic to give strength and stability to the denture. After curing, deflasking was done from the flask, and any excess acrylic was trimmed away.

Post-curing, the thermocol was removed carefully by making escape holes in both the obturator and ridge areas through flushing self-cure monomer through them. These holes allowed easy removal of the Thermocol, and the holes were later sealed with self-cure acrylic resin to restore structural integrity to the denture. This achieved a near-balanced prosthesis with optimum function and minimum weight.

**Figure 8:**
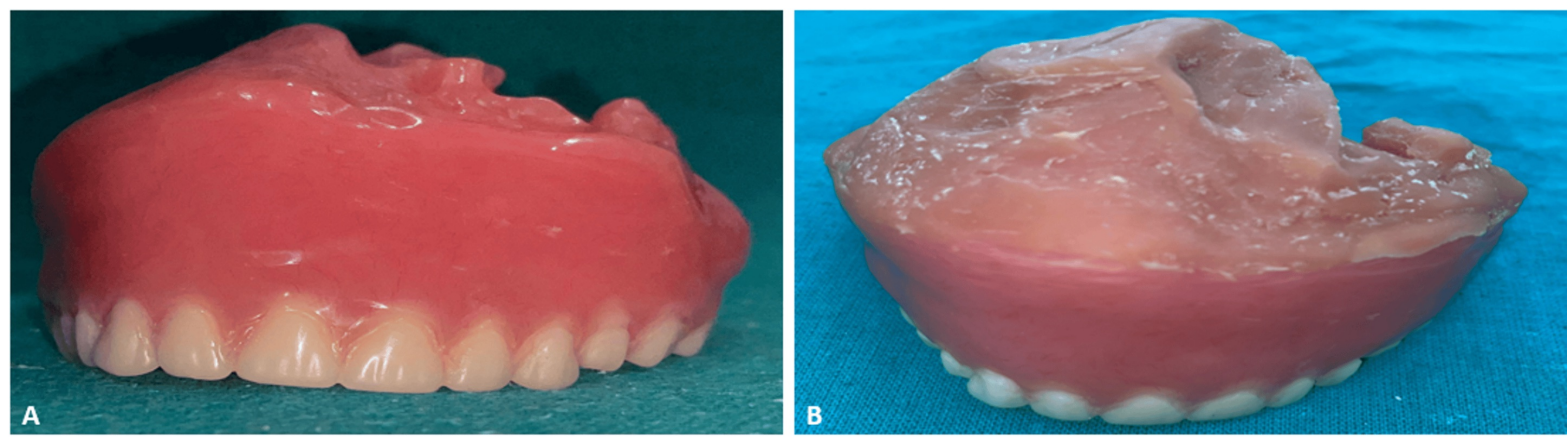
Maxillary complete denture with hollow bulb obturator: ex situ A: Finished complete denture; B: Complete denture with soft relining material

The smoothing of rough edges and the finishing of the denture with optimum fit were the last steps. Thus, both functional and aesthetic properties were checked to evaluate the final prosthesis after curing, which had a hollow bulb obturator. The hollow bulb obturator was expected to seal the palatal defect, thus keeping the patient comfortable while improving the speech and swallowing functions (Figure 8).

Relining of the finished complete denture was carried out using Detax Molloplast B (Detax GmbH & Co. KG, Ettlingen, Germany), which is a permanent soft relining material designed for use in dental laboratories. This one-component silicone material offers both heat-curing and microwave-polymerizing options, providing flexibility in processing. This material was chosen to provide maximum comfort and fit of finished dentures in the sensitive or atrophic areas of the ridge because it thus cushions the denture and gives better adaptation to the oral tissues and better retention. This has caused the tissues to experience greater comfort during extended wear and has helped prevent irritation of soft tissues (Figure 8).

**Figure 9:**
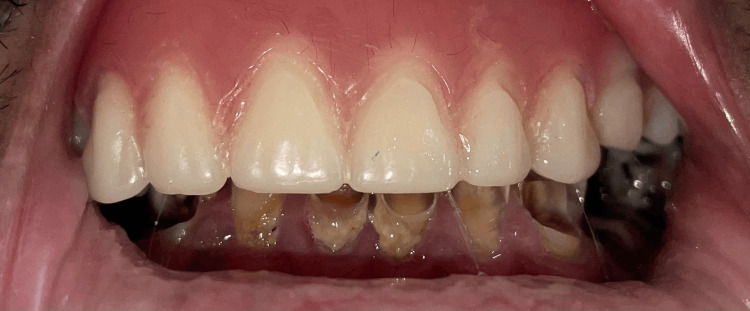
Maxillary complete denture with hollow bulb obturator: in situ

The maxillary complete denture with a hollow-bulb obturator was placed for the first time in the mouth of the patient. Immediately after placing the denture, the fit was checked carefully, along with the occlusion and the rest of the anatomical structure of the patient. The fit problems were immediately located from the discomfort or incomplete contact points, and appropriate changes were made to their records for optimum retention, stability, and comfort. It included alteration of the denture flange, occlusion, and the hollow obturator portion. Alterations were made to ensure that the obturator seals the palatal defect competently to enable better speech and swallowing function. The patient was completely satisfied with the condition after comprehensive assessment and adjustment procedures, indicating the comfort and functional efficiency of the prosthesis (Figure 9).

After the insertion of the complete denture with a closed hollow bulb obturator, the first follow-up appointment should be scheduled 24 hours after insertion, the second 72 hours later, and the third follow-up one week after insertion. These early follow-ups allow for the adjustment of any pressure areas or discomfort. Subsequent appointments should be scheduled as needed for further denture adjustments. It is crucial to gradually increase wearing time while avoiding hard foods during the initial period. Daily cleaning with a soft brush and mild soap, along with soaking the denture overnight in water to prevent drying out, is recommended. Regular follow-up visits will ensure continued comfort and optimal fit, with relining or rebasing as necessary due to changes in oral tissues. Dentures should be carefully handled, stored in water when not in use, and cleaned with denture-specific solutions to avoid damage.

There were several regular appointments to follow up with the patient to examine the performance and comfort of the maxillary complete denture with the hollow bulb obturator. At the first follow-up visit, approximately a week after insertion, speech and swallowing improved tremendously, with no major discomfort noted by the patient. Adjustments were made to the occlusion and fit of the denture for better retention and general comfort.

Subsequent appointments were for assessments such as the soft lining (Molloplast B) to ensure its cushioning effect. The prosthesis was then checked for wear or evidence of irritation to the mucosal tissues. The patient, too, received the education necessary to care for her denture in terms of hygiene, with a clear emphasis on everyday cleaning and periodic relining to achieve long-term success. 

At the end of six months, the patient had continued to enjoy satisfactory outcomes with the denture, and no adverse effects or problems were reported. The prosthesis remained stable, had good retention, and was free from any evidence of significant irritation or discomfort to the tissues. The patient was advised to come back for annual follow-up visits to check on the integrity of the prosthesis and make the necessary adjustments when needed.

## Discussion

The presentation case demonstrates the successful restoration of a patient with a maxillary complete denture incorporating a closed hollow bulb obturator, which plays a significant role in maxillary defect management. The introduction of the hollow bulb obturator provides utmost functionality and aesthetics to patients with palatal defects due to congenital anomalies or acquired trauma. In order to minimize the weight of the prosthesis and ensure maximum comfort for the patients, the hollow design of the obturator allows greater retention and stability with respect to the denture [7].

A systematic method was followed for the complete denture construction that included the initial impression making, custom tray fabrication, bite registration, and arrangement of teeth. These various procedures in the acrylization process, from flasking to wax removal, packing, and curing, were vital in the development of a durable and stable prosthesis [8]. The use of thermocol during the flasking process to create a hollow space in the obturator and the alveolar ridge contributed to the making of a lightweight functional denture [9]. The thermocol was then removed, flushed with self-cure monomer, and the escape holes were sealed off with self-cure acrylic resin to restore the structural integrity of the denture.

The insertion and adjustment process of the complete denture was a crucial step in achieving the final functional and aesthetic effect. Adjustments done post-insertion are part of the regular denture-fitting procedure to allow for additional fine-tuning of occlusion and adaptation to the soft tissues. In this context, they were necessary for proper fit, comfort, and retention, major contributors to patient satisfaction and long-term success of the prosthesis. The further application of Molloplast B for soft relining added to the patient's comfort with a cushioning effect and better adaptation to the mucosal tissues [10-11].

This treatment's merit indicates that a personal and systematic approach to the fabrication of dentures has to be given importance in palatal defects. The bulb obturator has served not only as an aid in speech and swallowing but also increased the overall quality of life for the patient by serving as a more comfortable and retentive prosthesis [12].

## Conclusions

This case highlights the successful prosthodontic rehabilitation of a patient using a maxillary complete denture with a closed hollow bulb obturator. Special attention was given during fabrication to create a lightweight and comfortable prosthesis by incorporating thermocol as the core material within the obturator. The design contributed to improved support by optimizing tissue contact and anatomical coverage, while the hollow configuration reduced overall weight, thereby enhancing prosthesis stability. Post-insertion adjustments significantly improved retention, fit, and patient satisfaction. To further enhance mucosal adaptation and long-term comfort, soft relining was carried out using Molloplast B. Functionally, the hollow bulb obturator provided an effective seal of the palatal defect, restoring essential functions such as speech and swallowing.

The case depicts a personalized and systematic approach toward prosthetic rehabilitation of palatal defect patients. The result shows how a hollow bulb obturator can improve the quality of life for patients who need complex maxillofacial prostheses. Further studies on the long-term effects of such prostheses will lead to new developments in the techniques and materials used for their fabrication toward better patient outcomes.
